# Supplementary material for: In Vivo Tracking and 3D Mapping of Cell Death in Regeneration and Cancer Using Trypan Blue
Source: Cells. 2024 Aug 20;13(16):1379. doi: 10.3390/cells13161379 (PMC11352400; doi:10.3390/cells13161379)
Supplement: Supplementary file 1 [file cells-13-01379-s001.zip › Procel Supp Figure S2.pdf]

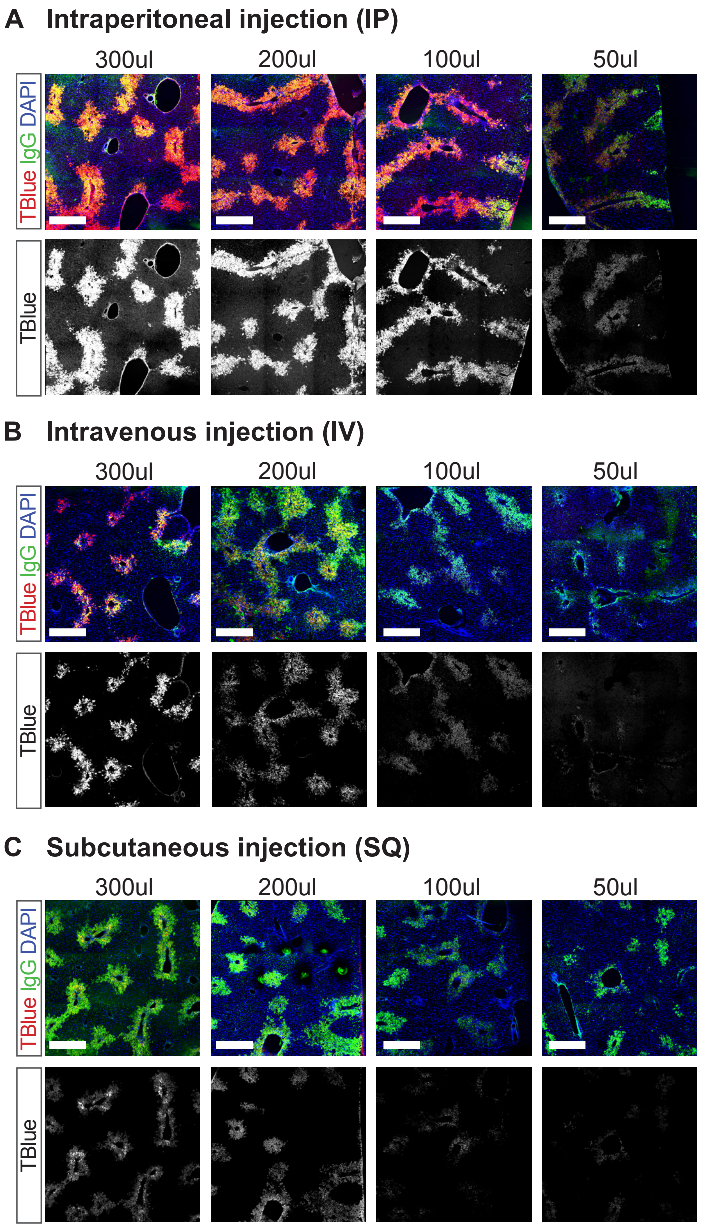

Supplementary Figure S2
