# Supplementary figures and images for: In Vivo Tracking and 3D Mapping of Cell Death in Regeneration and Cancer Using Trypan Blue
Source: Cells. 2024 Aug 20;13(16):1379. doi: 10.3390/cells13161379 (PMC11352400; doi:10.3390/cells13161379)

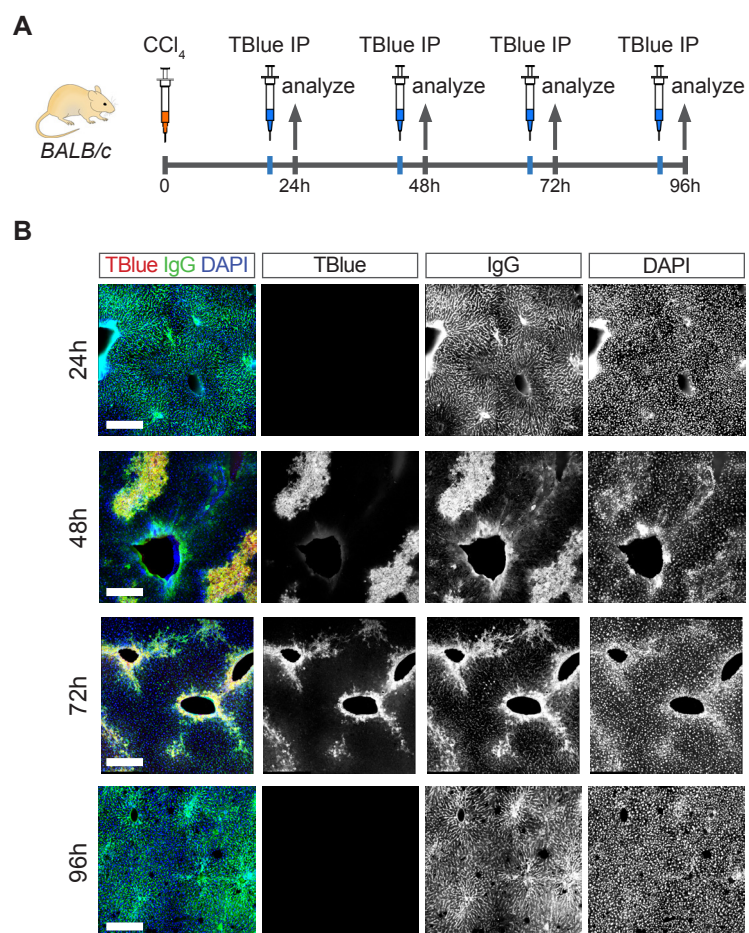

Supp Figure S1

Supplement: Supplementary file 1 [file cells-13-01379-s001.zip › Procel Supp Figure S1.pdf]

A

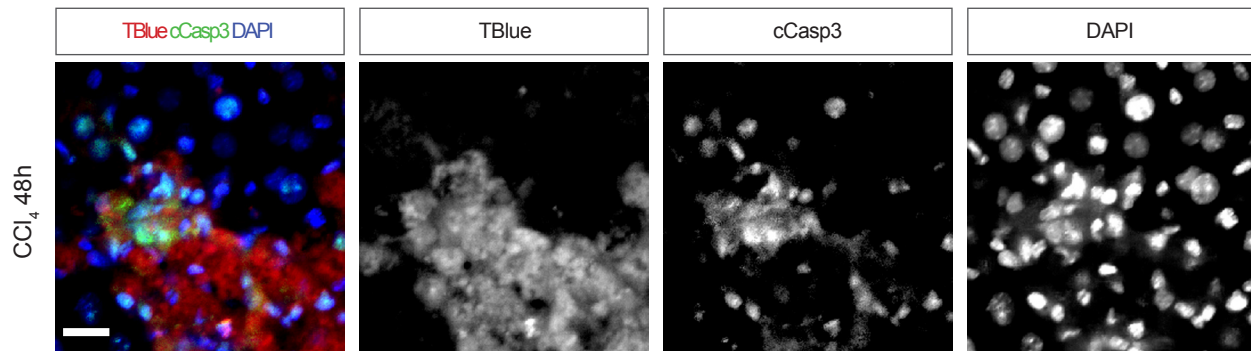

Supplementary Figure S3

Supplement: Supplementary file 1 [file cells-13-01379-s001.zip › Procel supp Figure S3.pdf]

**A**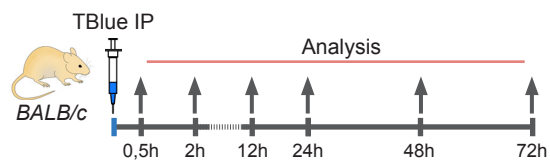**B**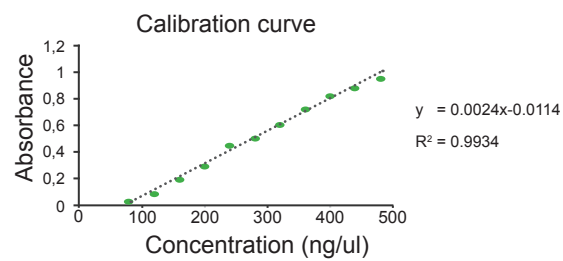**C**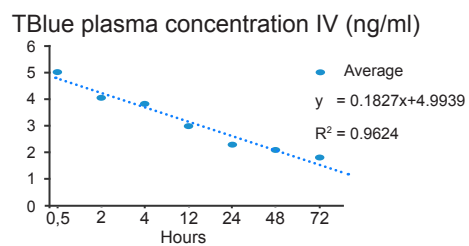**D**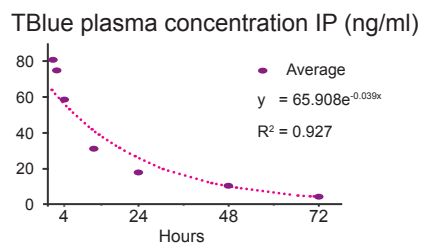

Supplementary Figure S4

Supplement: Supplementary file 1 [file cells-13-01379-s001.zip › Procel Supp Figure S4.pdf]

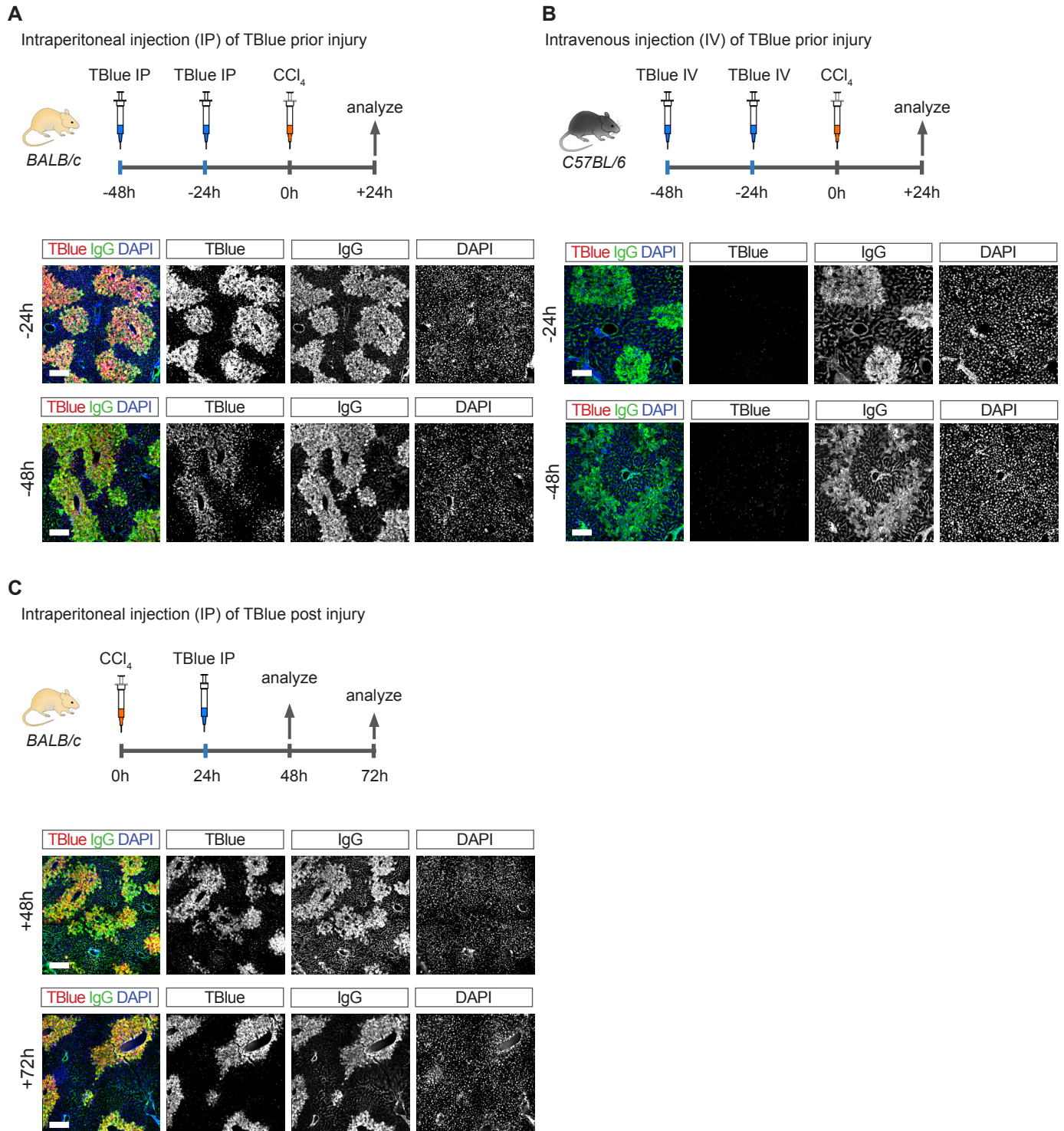

Supplementary Figure S5

Supplement: Supplementary file 1 [file cells-13-01379-s001.zip › Procel Supp Figure S5.pdf]

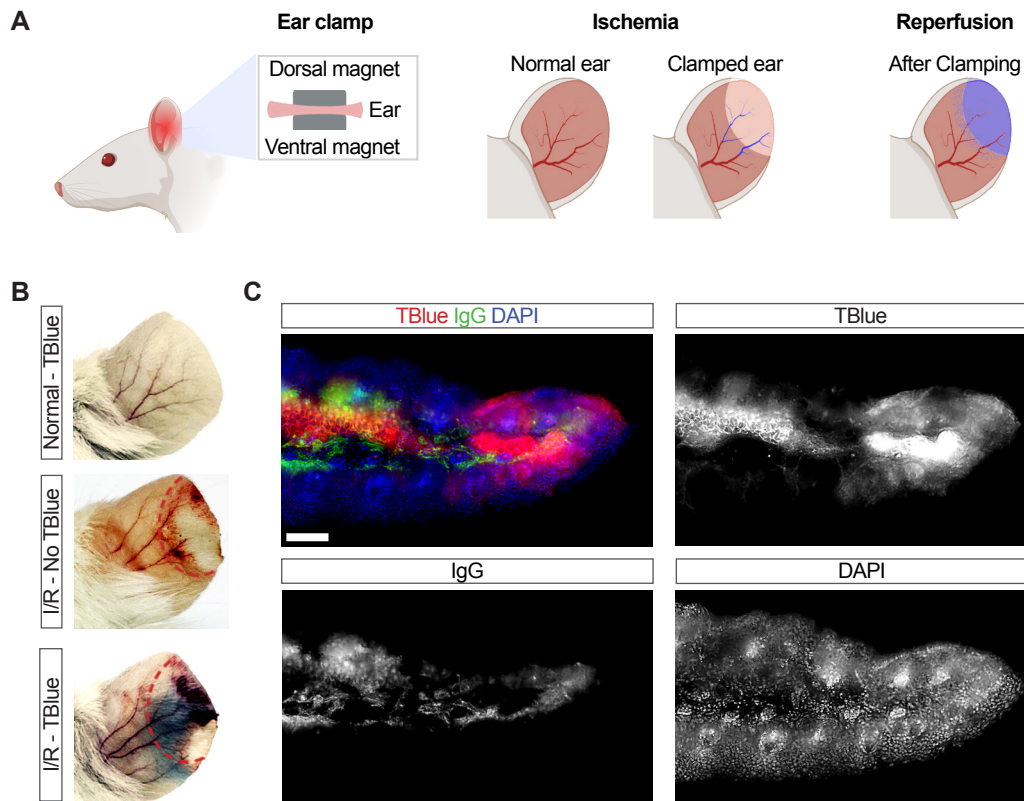

Supplementary Figure S6

Supplement: Supplementary file 1 [file cells-13-01379-s001.zip › Procel supp Figure S6.pdf]

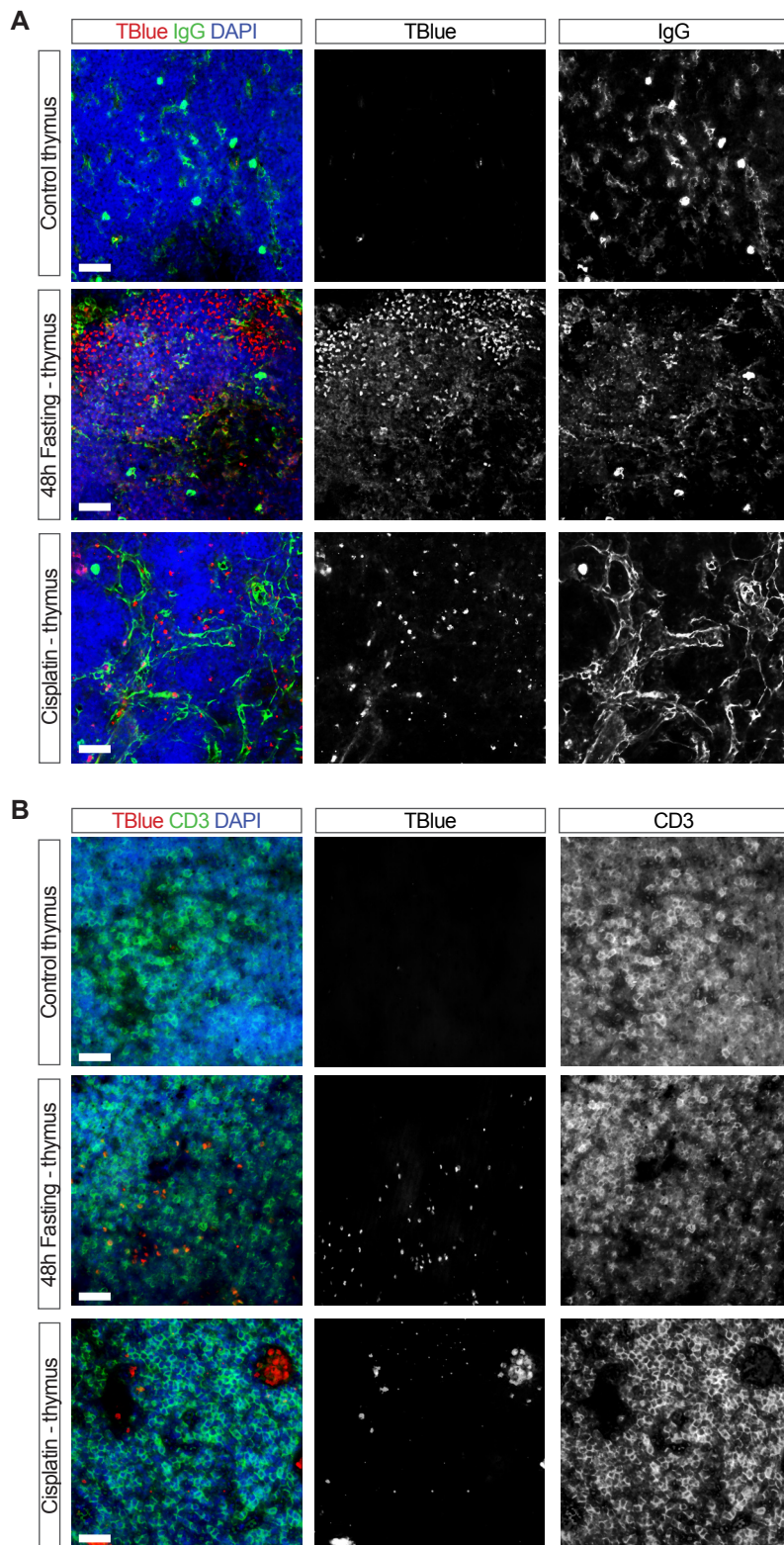

Supplementary Figure S7

Supplement: Supplementary file 1 [file cells-13-01379-s001.zip › Procel Supp Figure S7.pdf]
